# The PTI1-like kinase ZmPti1a from maize (*Zea mays *L.) co-localizes with callose at the plasma membrane of pollen and facilitates a competitive advantage to the male gametophyte

**DOI:** 10.1186/1471-2229-6-22

**Published:** 2006-10-06

**Authors:** Markus M Herrmann, Sheena Pinto, Jantjeline Kluth, Udo Wienand, René Lorbiecke

**Affiliations:** 1 Biozentrum Klein-Flottbek und Botanischer Garten, Universität Hamburg, Ohnhorststrasse 18, 22609 Hamburg, Germany; 2 Deutsches Krebsforschungszentrum, Im Neuenheimer Feld 580, 69120 Heidelberg, Germany

## Abstract

**Background:**

The tomato kinase Pto confers resistance to bacterial speck disease caused by *Pseudomonas syringae *pv. *tomato *in a gene for gene manner. Upon recognition of specific avirulence factors the Pto kinase activates multiple signal transduction pathways culminating in induction of pathogen defense. The soluble cytoplasmic serine/threonine kinase Pti1 is one target of Pto phosphorylation and is involved in the hypersensitive response (HR) reaction. However, a clear role of Pti1 in plant pathogen resistance is uncertain. So far, no Pti1 homologues from monocotyledonous species have been studied.

**Results:**

Here we report the identification and molecular analysis of four Pti1-like kinases from maize (ZmPti1a, -b, -c, -d). These kinase genes showed tissue-specific expression and their corresponding proteins were targeted to different cellular compartments. Sequence similarity, expression pattern and cellular localization of ZmPti1b suggested that this gene is a putative orthologue of Pti1 from tomato. In contrast, ZmPti1a was specifically expressed in pollen and sequestered to the plasma membrane, evidently owing to N-terminal modification by myristoylation and/or S-acylation. The ZmPti1a:GFP fusion protein was not evenly distributed at the pollen plasma membrane but accumulated as an annulus-like structure which co-localized with callose (1,3-β-glucan) deposition. In addition, co-localization of ZmPti1a and callose was observed during stages of pollen mitosis I and pollen tube germination. Maize plants in which ZmPti1a expression was silenced by RNA interference (RNAi) produced pollen with decreased competitive ability. Hence, our data provide evidence that ZmPti1a plays an important part in a signalling pathway that accelerates pollen performance and male fitness.

**Conclusion:**

ZmPti1a from maize is involved in pollen-specific processes during the progamic phase of reproduction, probably in crucial signalling processes associated with regions of callose deposition. Pollen-sporophyte interactions and pathogen induced HR show certain similarities. For example, HR has been shown to be associated with cell wall reinforcement through callose deposition. Hence, it is hypothesized that Pti1 kinases from maize act as general components in evolutionary conserved signalling processes associated with callose, however during different developmental programs and in different tissue types.

## Background

Protein kinases in plants have been found to be involved in basic features of plant defense and plant fertilization. Increasing knowledge about the underlying molecular mechanisms suggests several parallels between both processes [1-3]. Plant-pathogen recognition has been studied extensively in tomato in which gene for gene resistance against certain *Pseudomonas syringae *pv. *tomato *strains is conferred by the serine/threonine kinase Pto. Upon recognition of bacterial avirulence factors, Pto acts in concert with the Prf protein resulting in the activation of multiple signal transduction pathways culminating in the induction of defense responses including HR [4]. Several Pto-interacting (Pti) proteins were identified to act in Pto-mediated signal transduction including the protein kinase Pti1 and three transcription factors (Pti4/5/6), respectively [5,6]. Pti1 (here referred to as SlPti1 for clarity reasons) is a cytoplasmic protein kinase capable of autophosphorylation *in vitro *[5] and moreover also be phosphorylated by Pto. Tobacco plants over-expressing S1Pti show enhanced HR in leaves in response to avirulence factor treatment indicating a functional role of SlPti1 in Pto-mediated disease response [5]. However, a precise role of SlPti1 in plant pathogen resistance has remained unclear, owing to functional redundancy of different/additional Pti1 kinases. Three SlPti1 homologous kinases have been cloned from soybean [7,8], sPti1a, sPti1b and GmPti1. The former two do not display *in vitro *autophosphorylation activity [7], whereas the latter, GmPti1, possesses autophosphorylation activity. *GmPti1 *gene expression was found to accelerate in response to wounding and salicylic acid treatment in seedling leaves [8]. These findings suggest different Pti1-like kinases to possess different properties and biological functions in plants.

Cell-cell recognition and signal response reactions during plant-pathogen interaction are thought to be molecularly related to certain steps of plant reproduction, e.g. pollen-pistil recognition, compatibility reactions, and pollen tube growth. In studies of the genetic and molecular basis of pollen development and function more than 150 pollen-expressed genes from more than 28 species have been identified [9-11]. Classification of pollen expressed genes identified a high number of genes which are involved in signal transduction. Many of these genes encode putative protein kinases [10,12,13]. Accordingly, leucine-rich repeat (LRR) Ser/Thr-type plant receptor kinases (PRK) LePRK1 to 3 from tomato and several interacting proteins like KPP, LAT52 and LeSHY have already been attributed to signaling processes during pollen tube growth [14-17].

Mutations of a number of such gametophytically important genes often result in altered Mendelian segregation ratios due to an abolished or reduced transmission of a linked marker through pollen. Such genes include SEC8, ROP2, LIMPET POLLEN and TTD genes [17-21]. Most of these mutations cause obvious defects in the pollen grain and affect early stages of pollen development. In contrast, only few mutations are known that are transmitted through the male at low frequencies but cause no obvious defects in pollen morphology. These genes appear to affect more pollen competitiveness rather than development, e.g. TTD41 and ROP2 [18,21].

In this study we report the identification and molecular analyses of four Pti1 kinases from maize (ZmPti1a, -b, -c, -d). The genes were expressed in different tissues and showed different subcellular localizations. Phylogenetic analysis revealed the existence of three conserved Pti1 kinase subgroups in higher plants. Based on its sequence similarity, expression profile and subcellular localization ZmPti1b was suggested to be a putative SlPti1 ortholog. In contrast, the functional kinase ZmPti1a was specific to pollen and targeted to the plasma membrane, evidently owing to N-terminal acylation. ZmPti1a co-localizes with regions of callose deposition at stages of pollen maturation and germination. Silencing of the ZmPti1a gene resulted in a significant decrease in the competitive ability of pollen. These findings provide evidences of ZmPti1a to play an important role in influencing pollen fitness.

Our data further suggest that Pti1 kinases from maize act in various tissues and in different but mechanistically conserved plant response pathways which likely involve similar signals and/or signal transduction molecules.

## Results

### Pti1-like kinases of maize

**Figure 1:**
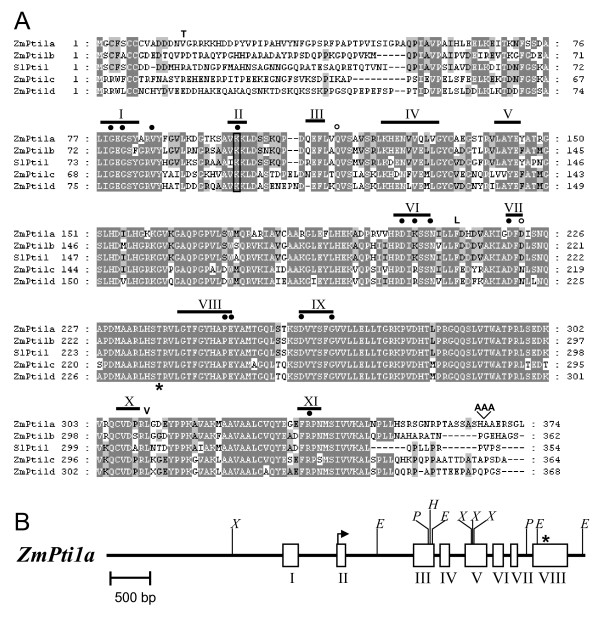
**Similarity and predicted genomic structure of ZmPti1a**. **(A) **Alignment of Pti1 kinases from maize with SlPti1 from tomato. Amino acids identical in at least three of the sequences are highlighted in grey. The 11 canonical subdomains conserved in serine/threonine kinases are indicated with Roman numerals. Invariant residues common to the majority of protein kinases are marked with black dots. Invariant residues that are conserved in other protein kinases but not in Pti1 kinases are marked with open circles. The highly conserved lysine residue in subdomain II which is required for activity in SlPti1 and most protein kinases is boxed. Threonine 233 has been identified as the major site of SlPti1 phosphorylation by SlPto and is marked with an asterisk. Amino acids which differ between ZmPti1a and the deduced protein sequence of the second cloned ZmPti1a cDNA [GenBank:AY554282] are indicated above the sequences. **(B) **Genomic locus and restriction map of the *ZmPti1a *gene. Exons are indicated as boxes with Roman numerals. Start and stop of the open reading frame are marked with an arrow and asterisk, respectively. E, *Eco*RI; H, *Hin*dIII; P, *Pst*I, X, *XhoI*.

A 217 bp partial cDNA of *ZmPti1a *was cloned in a molecular approach with the aim to identify genes that are specifically expressed in maize pollen. Using this clone as a hybridization probe, two nearly identical 1.6 kb full-length cDNAs [GenBank:AY554281, GenBank:AY554282] were isolated from a λ-cDNA library of *in vitro *germinated pollen from *white pollen *(*whp*) plants [22] expressing the *c2 *gene. Both cDNAs probably represent different alleles of the same gene. The cDNA clone AY554281 was further analyzed in this study. AY554281 contains an open reading frame (ORF) of 1122 bp, a 207 bp 5' untranslated region and a 304 bp 3' untranslated region including a poly(A)^+ ^tail. The putative protein of AY554281 is 374 amino acids (aa) in length with a molecular mass of 40.8 kDa (Fig. 1A). Database search revealed 69% identity and 75% similarity to the *Pto-interactor 1 *(*Pti1*) protein kinase of *Solanum lycopersicum *[5]. Therefore the cloned gene was named *Zea mays Pti1a (ZmPti1a)*. The putative catalytic kinase domain of ZmPti1a starts approximately 75 aa after the first methionine and contains 11 canonical subdomains that are typical of serine/threonine kinases (Fig. 1A; [23]). Out of the 15 invariant amino acid residues common to the majority of protein kinases, 13 were found to be conserved in ZmPti1a (Fig. 1A). A glutamine in subdomain III is substituted with a glutamate at position 115 and a conserved glycine in subdomain VII is substituted with an aspartate at position 222. Identical substitutions are present in the kinase SlPti1 [5] suggesting that *ZmPti1a *is also a functional kinase. A corresponding full-length genomic clone [Genbank:AY554283] spanning the entire transcribed region as well as 2.2 kb of the promoter of *ZmPti1a *was isolated from a λ-phage library of the maize inbred LC by plaque screening and inverse PCR. The gene consists of 8 exons and 7 introns (Fig. 1B). The nucleotide sequence of the deduced transcribed region was found to be nearly identical to the previously cloned cDNAs with the exception of line specific single nucleotide polymorphisms that changed three aa in less conserved regions of the deduced protein. An insertion of 9 bp resulted in the addition of three alanine residues in the c-terminus (Fig. 1A). The proposed translation start is located in exon 2. Hybridizing bands in genomic Southern analyses with probes specific for the promoter, 5'-UTR, ORF, and 3'-UTR of *ZmPti1a *correlated well with the predicted restriction patterns of the cloned gene and suggested that *ZmPti1a *is a single copy gene (data not shown).

Database search led to the identification of additional ESTs coding for *ZmPti1a *homologues from maize. These sequences were found to be well conserved at the nucleotide level (41 to 52%), and even more conserved at the the protein level (71 to 78%). Corresponding ORF and 3' UTRs were amplified by RT-PCR from lines A188 and LC, respectively. All cloned sequences were identical to their corresponding EST with the exception of few line specific SNPs. Accordingly, these sequences were named *ZmPti1b *[Genbank:DQ647388], *ZmPti1c *[Genbank: DQ647389], and *ZmPti1d *[Genbank: DQ647390], respectively. An EST clone [Genbank:AY708048] which resembles *ZmPti1c *was annotated previously as a salt-inducible putative serine/threonine/tyrosine kinase (Zou *et al.*, unpublished data). Data mining of genomic BAC and MAGI sequences containing *ZmPti1b *and -*d *indicated that the corresponding genes possess nearly identical exon/intron structures as compared to *ZmPti1a *(data not shown). This indicates that the maize *Pti1 *gene family most likely originates from a single ancestor gene. Out of the four putative ZmPti1 kinases, ZmPti1b showed highest protein similarity to Pti1 from tomato (77% identity, 85% similarity). All ZmPti1 proteins possess conserved kinase catalytic domains. However, their N – and C-terminal regions are highly variable and only some Pti1 kinases, including ZmPti1a, were predicted to contain a putative myristoylation signal at their N-termini. Such protein modifications in which the saturated fatty acid myristate is covalently but reversibly attached to an N-terminal Gly after co-translational cleavage of the first Met residue can fulfill several functions, e.g. mediating membrane association.

### Phylogenetic relationship of ZmPti kinases

**Figure 2:**
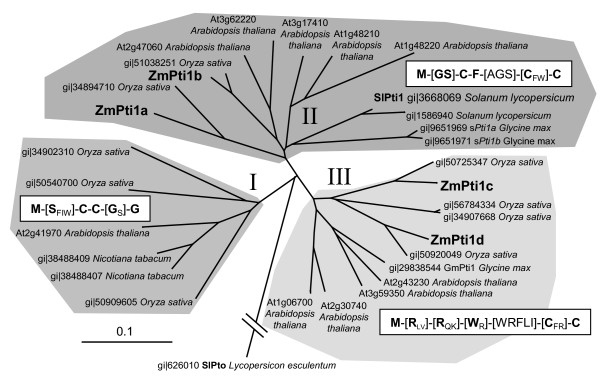
**Phylogenetic analysis of ZmPti1 kinases**. Similarity and phylogenetic relationship of Pti1 proteins from maize, rice, tobacco, soybean and tomato were calculated using *ClustalX *and visualized using *Treeview*. SlPto [gi 626010/pir:A49332] was used as the outgroup. Consensus sequences of the N-termini are given for each subgroup. Highly conserved residues are indicated in bold. Ambiguities are given in brackets with residues of high appearance in bold and of less appearance in subscribed letters.

Phylogenetic comparison of ZmPti1 proteins from maize and putative Pti1 kinases from other plants indicated three major Pti1 subgroups in angiosperms (I, II & III) with the known maize proteins belonging to subgroups II and III, respectively (Fig 2). Each subfamily possesses a conserved N-terminal domain with a specific consensus sequence and consists of proteins from mono – as well as dicotyledonous species. The N-terminal domains are rich in polar or aromatic residues and contain at least two conserved cysteines. Some of the kinases, e.g. ZmPti1a, sPti1a, sPti1b and At3g17410 are predicted to contain a putative N-terminal myristoylation signal. Gene organization of most of the Pti1 kinases from *Arabidopsis thaliana *were found to be similar to that of *ZmPti1a*, i.e. 8 exons and a predicted translation start in exon 2 (data not shown).

Based on these findings, *Pti1 *genes appear to represent an ancient kinase family in higher plants. Amino acid sequences of the different N-terminal regions are conserved in a broad spectrum of monocotyledonous and dicotyledonous species (Fig. 2). Thus, it is feasible to speculate that the conserved N-terminal motifs of the different Pti1 subfamilies were retained during evolution because of specific relevant biological functions.

### ZmPti1 proteins localize to different subcellular compartments

**Figure 3:**
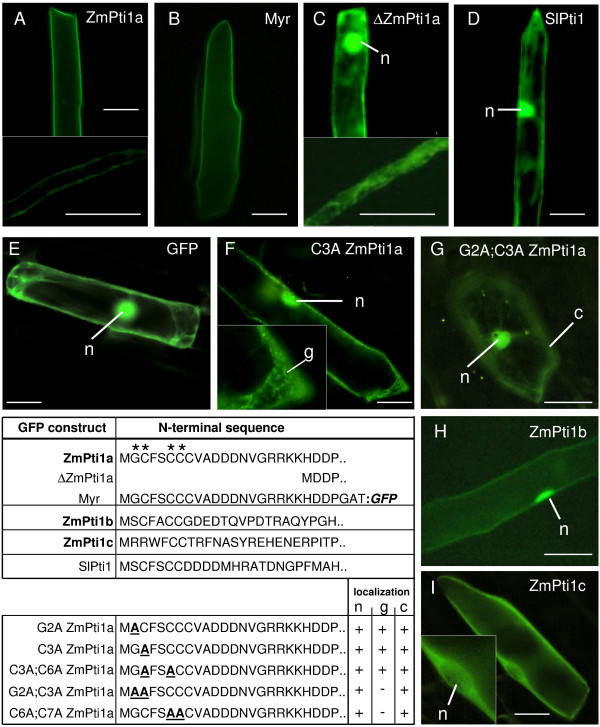
**Transient expression of GFP fusion constructs**. Wild type ZmPti1a, ZmPti1b, ZmPti1c, SlPti1 and N-terminal mutants of ZmPti1a were transiently expressed as C-terminal GFP fusion proteins in onion epidermal cells and in *in vitro *germinating pollen, respectively. Schematic representations of wild type and mutant GFP fusions are depicted in the table. Amino acids being potential targets for N-terminal modification are marked with asterisks above the ZmPti1a wild type sequence. For mutant constructs the subcellular localization is symbolized in the table; nucleus (n); cytoplasmic granules (g); cytoplasm (c). **(A) **Wild type ZmPti1a. Top panel: onion epidermal cell; bottom panel: pollen tube **(B) **24 aa of the ZmPti1a N-terminus fused to GFP. **(C) **N-terminally truncated ZmPti1a. Top panel: onion epidermal cell; bottom panel: pollen tube **(D) **Wild type SlPti1 from tomato. **(E) **GFP control. **(F) **ZmPti1a containing Cys3 → Ala3 mutation. **(G) **ZmPti1a containing Gly2 → Ala2 and Cys3 → Ala3 mutation. **(H) **Wild type ZmPti1b. **(I) **Wild type ZmPti1c. Scale bars = 50 μm.

To investigate the subcellular localization of ZmPti1 proteins *in situ*, we transiently expressed in-frame coding sequences of ZmPti1 kinases fused to green fluorescent protein (GFP) in onion epidermal cells and in *in vitro *germinating pollen, respectively. When expressed under control of the ubiquitin promoter, ZmPti1a:GFP was targeted to the cell periphery suggesting ZmPti1a to localize to the plasma membrane (Fig 3A). This pattern was clearly different from that observed when GFP was expressed alone (Fig 3E). Association of ZmPti1a:GFP with the plasma membrane was also proven by confocal laser scanning microscopy (data not shown). Twenty-four amino acids of the ZmPti1a N-terminus were found to be sufficient to target GFP entirely to the cell periphery (Myr:GFP, Fig. 3B). Truncation of twenty amino acids at the N-terminus of ZmPti1a abolished cell periphery targeting coinciding with cytoplasmic and nuclear localization of the fusion protein (ΔZmPti1a, Fig 3C). Identical results were observed for these three ZmPti1a fusion constructs when expressed ectopically in stably transformed maize plants (Fig. 6 and data not shown). These findings are in agreement with the assumption that ZmPti1a is targeted to the plasma membrane by N-terminal acylation, likely myristoylation.

To study the structural basis of ZmPti1a being targeted to the plasma membrane in detail, potential myristoylation and/or palmitoylation sites, i.e. Gly2/Cys3 and Cys6/Cys7 were subjected to site-directed mutagenesis (Table Fig. 3). Conjugation of myristate to proteins is absolutely dependent on a glycine residue at position 2.

Exchange of Gly2 or Cys3 with Ala prevented targeting of the ZmPt1a:GFP fusion to the cell periphery. Instead, GFP fluorescence appeared in the nucleus and as small cytoplasmic granules (Fig. 3F and data not shown). The same GFP pattern was seen when both, Cys3 and Cys6, were replaced by Ala (data not shown).

Combined replacement of the adjacent amino acids Gly2 and Cys3 with Ala residues also caused nuclear localization. However, GFP fluorescence was evenly distributed in the cytoplasm and no granules were observed (Fig. 3G). A similar distribution of GFP fluorescence was observed when Cys6 and Cys7 in the second motif were replaced with alanine residues (data not shown).

These results indicate that combined mutation of single residues in each of the two motifs (Gly2/Cys3 or Cys6/Cys7) resulted in GFP fluorescence associated with cytoplasmic granules. This localization pattern might reflect an imperfect targeting or mistargeting of mutated ZmPti1a to membranes. Combined replacement of both adjacent residues in either one of the two motifs seems to strengthen mistargeting and completely prevents ZmPti1a membrane association.

ZmPti1b, c and d from maize and SlPti1 from tomato naturally lack a Gly2 residue that would serve as a potential target site of myristoylation (Fig. 1A). Accordingly, Zhou *et al*. [5] predicted the tomato SlPti1 to be a cytoplasmic kinase. Expression of a SlPti1:GFP fusion protein confirmed the theoretical prediction however, additionally revealed a nuclear localization (Fig. 3D). A similar localization pattern was observed for the GFP fusion of ZmPti1b, which is the closest SlPti1-homologue from maize (Fig. 3H). By contrast, GFP fusions of ZmPti1c and ZmPti1d appeared only in the cytoplasm but not in the nucleus (Fig. 3I, data not shown). Both of these sub-group III proteins contain a conserved N-terminal pair of arginine residues instead of a myristoylation signal. Taken together, ZmPti1 family members show a differential competence for plasma membrane association, evidently owing to their varying susceptibility to N-terminal myristoylation and/or S-acylation.

### *ZmPti1 *genes are expressed in different maize tissues

**Figure 4:**
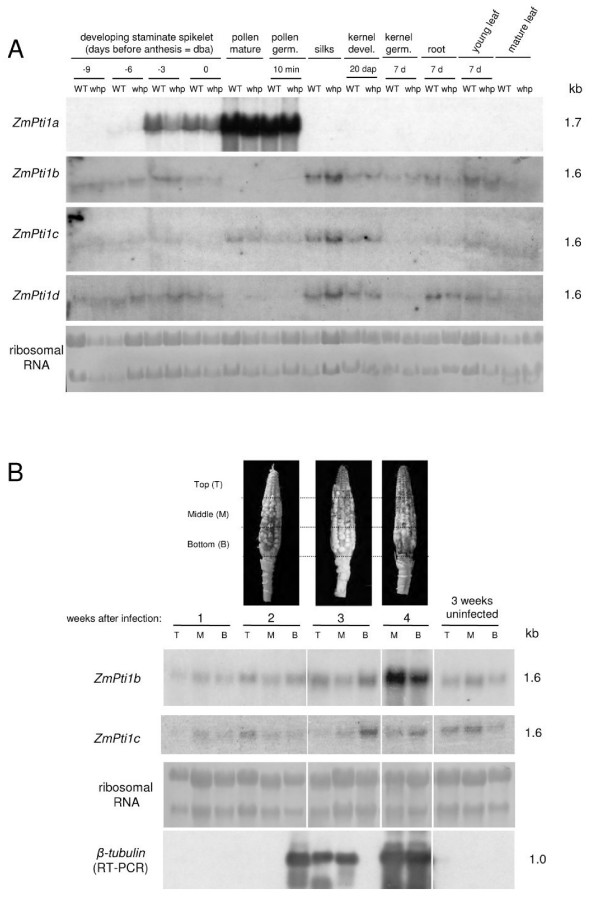
**Expression of *ZmPti1 genes *in various tissues at different developmental stages of maize**. **(A) **Expression of *ZmPti1 *genes was analyzed in the sterile flavonol-deficient *whp *maize line and its corresponding wild type (WT) in developing staminate spikelets 9 dba (-9), 6 dba (-6), 3 dba (-3) and at anthesis (0) in mature pollen isolated from spikelets at anthesis (pollen mature), in pollen germinated *in vitro *for 10 min (pollen germ.), in silks, developing kernels 20 days after pollination (kernel dev. 20 dap), in kernels 7 days after germination (kernel germ.), in roots and leaves of 7-d-old seedlings and in mature leaves. Methylene blue stained ribosomal RNA is shown as loading control. **(B) **Expression of *ZmPti1 *genes in *Fusarium graminearum *infected maize cobs. Pathogen infected maize cobs of line A188 were harvested over a period of four weeks after infection and of a mock infected cob (3 weeks uninfected). RNA was isolated from kernels of cobs and divided into top (T) middle (M) and bottom (B). Corresponding cobs are shown at the top of the figure. 20 μg of total RNA of each probe were utilized for Northern blotting and subsequently hybridized to probes specific to *ZmPti1b *and *ZmPti1c*, respectively. Methylene blue stained ribosomal RNA served as loading control of the gel. Transcripts of a constitutively expressed *Fusarium *β-tubulin gene [60] were amplified by semi-quantitative RT-PCR from the same RNA probes, blotted and hybridized to a β-tubulin specific probe. The amount of amplified cDNAs served as an indicator of the severity of pathogen infection (β-tubulin RT-PCR).

Gene regulation of the four identified members of the *ZmPti1 *family was studied during sporophytic and gametophytic development of maize. We used both, a wild-type and the *whp *maize line of which the latter produces sterile pollen due to the lack of flavonol synthesis. Northern blot analysis revealed a tissue specific expression pattern for *ZmPti1a *with extremely high mRNA levels in staminate spikelets ('male flower') of male inflorescences and in pollen. *ZmPti1a *transcript increased strongly during flower development between 6 days before anthesis (dba) and anthesis (Fig. 4A). Even higher transcript amounts were detected in isolated mature pollen harvested at anthesis. Since mature pollen and staminate spikelets at anthesis are at the same developmental stage, it is likely that *ZmPti1a *expression in spikelets is mainly due to its specific expression in the enclosed pollen rather than in the surrounding sporophytic tissue.

In contrast to *ZmPti1a, ZmPti1b*, *c *and *d *were expressed at low levels but in all sporophytic tissue types analyzed. Only transcripts of *ZmPti1c *could also be detected in mature and germinated pollen (Fig. 4A).

Because expression of *ZmPti1b *and *ZmPti1d *was absent in mature and germinated pollen, appearance of the corresponding transcripts in all stages of the male flower development may indicate that these genes are preferentially expressed in the sporophytic tissue of the male inflorescence.

In pollen, transcript amounts of *ZmPti1a *and *ZmPti1c *did not differ between the pollen-sterile mutant *whp *and its corresponding wild-type line (WT) indicating that pollen sterility, due to a lack in flavonoid biosynthesis, did not alter expressions of both *ZmPti1 *genes.

Taken together, these results showed that three members of the *ZmPti1 *family were similarly expressed in the sporophytic maize tissues with *ZmPti1c *also being expressed in pollen. *ZmPti1a *expression differed significantly because of its strong pollen specific expression.

### *ZmPti1b *transcript increase in maize kernels after pathogen infection

SlPti1 from tomato was shown to be involved in HR [5]. To investigate if some of the *ZmPti1 *genes play a role in pathogen defense, we studied the expression of *ZmPti1 *genes in developing maize kernels that were infected with the crop pathogen *Fusarium graminearum*. Maize cobs were infected two days after fertilization and harvested at different time points after infection. Because fungal infections usually proceed in diverse gradients on infected cobs, kernels were harvested from three regions i.e. top, middle and bottom of individual cobs.

The severeness of fungal infection in these samples was monitored based on the visual rating using the silk channel scale [24] and by RT-PCR detection of a fungal specific β-tubulin mRNA (Fig 4B.). The amounts of amplified β-tubulin cDNAs correlated perfectly with the phenotypically visible severity of fungal infection on the cobs.

Northern blot analysis revealed a four-fold enhanced *ZmPti1b *transcript level in infected kernels isolated from a cob possessing a disease severity 6 indicating 51%–75% of infection (Fig. 4B; 4 weeks after infection). No RNA could be extracted from kernels located at the top of this cob because of the advanced mode of fungal infection. Previous Northern experiments showed that *ZmPti1b *was constitutively expressed during kernel development (data not shown). Therefore, the accelerated *ZmPti1b *expression could be attributed to pathogen infection. No changes of *ZmPti1b *transcript levels were detected in kernels from an uninfected cob or from cobs with less severe disease patterns (Fig 4; 2 and 3 weeks after infection). Owing to the discrepancy of the highly variable mode of fungal infection no conclusions could be drawn from the experiment with respect to the putative time dependence of *ZmPti1b *induction during *Fusarium *infection. However, accelerated *ZmPti1b *expression upon *Fusarium *infection was proven in a second independent experiment (data not shown).

Because the experiments were conducted under non-sterile green house conditions it can not be excluded that weakening of the cob tissues after *Fusarium *infection was a result of additional pathogens, e.g. bacteria, which in turn may have triggered *ZmPti1b *expression. In contrast to *ZmPti1b*, expression of *ZmPti1c *and d was not significantly altered in this experiment.

Northern data provided evidence that at least one of the putative maize Pti1 kinases, ZmPti1b, may function in pathogen defense similar to what was previously shown for tomato SlPti1 [5]. Accordingly, ZmPti1b was identified to be the closest homologue of SlPti1 among the four identified ZmPti1 kinases (Fig. 1A and Fig. 2). Together with the phylogenic data, this finding supports the hypothesis that related Pti1 kinases could possess similar biological functions in different plant species.

### *ZmPti1a *encodes a functional protein kinase

**Figure 5:**
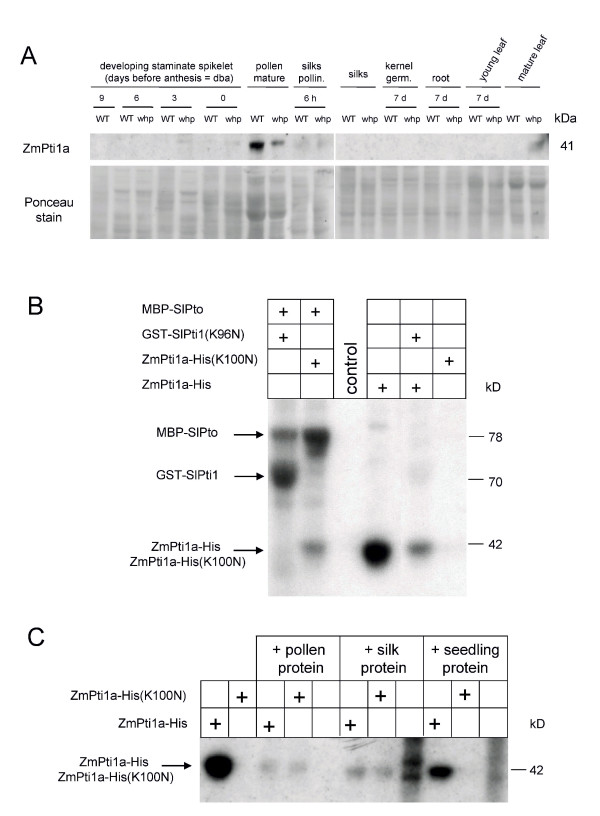
**ZmPti1a protein expression and kinase activity**. **(A) **Immunodetection of ZmPti1a protein in various tissues at different developmental stages of maize. Proteins from the sterile flavonol-deficient *whp *maize line and its corresponding wild type (WT) were size fractionated using PAGE (silks pollen, pollinated silks 6 h after pollination; see legend Fig. 3 for other tissues). Proteins were subjected to Western blot and detected using a polyclonal antibody raised against recombinant ZmPti1a. Ponceau stain of the blot is given as a control for loading of the gel. A strong band of the expected molecular size of the ZmPti1a protein of approximately 41 kDa is visible in extracts from mature pollen. Faint bands could be detected in protein of staminate spikelets at anthesis (0 dba) and pollinated silks after extended exposure times. **(B) **Autophosphorylation and cross-phosphorylation of ZmPti1a. Wild type ZmPti1a-His fusion protein, wild type MBP-SlPto, and the kinase-deficient mutants ZmPti1a-His(K100N) and GST-SlPti1(K96N) were over-expressed and purified in equal amounts from *E. coli*, using Ni-NTA magnetic beads, *GST-Bind-Resin *or *amylose resin*, respectively. Immobilized proteins were incubated alone or in pairs with [γ-^32^P]ATP in kinase buffer, separated by PAGE and exposed to X-ray film. Cross-phosphorylation of GST-SlPti1(K96N) by MBP-SlPto served as positive control. ZmPti1a is capable of autophosphorylation. The K100N mutation completely abolished autophosphorylation of ZmPti1a. ZmPti1a-His(K100N) is moderately phosphorylated by MBP-SlPto whereas ZmPti1a-His cannot phosphorylate GST-SlPti1. **(C) **Magnetocapture interaction kinase assay. Wild type ZmPti1a-His or mutant ZmPti1a-His(K100N) was immobilized on Ni-NTA magnetic beads and incubated with native protein extracts from pollen, silks or seedlings. ZmPti1 and bound proteins were collected by magnetic force, washed and subjected to kinase assays. Unloaded Ni-NTA magnetic beads were used as control. Proteins were separated by PAGE and exposed to X-ray film. Pollen and silk but not seedling extracts contained kinase activities capable of interaction with and cross-phosphorylating ZmPti1a-His(K100N).

Because of its unusually specific expression in pollen, further investigations were focused on the biological function of ZmPti1a. Western analysis of various maize tissues using a polyclonal antibody raised against bacterially expressed ZmPti1a detected a protein with the expected molecular size of 41 kDa in mature pollen (Fig 5A). Faint bands appeared in extracts from staminate spikelets at anthesis and pollinated silks after longer exposure times (data not shown). No such bands could be detected in other tissues indicating that ZmPti1a protein is specifically expressed in pollen. Hence, protein expression was shown to correlate well with the abundance of its corresponding mRNA.

**Figure 6:**
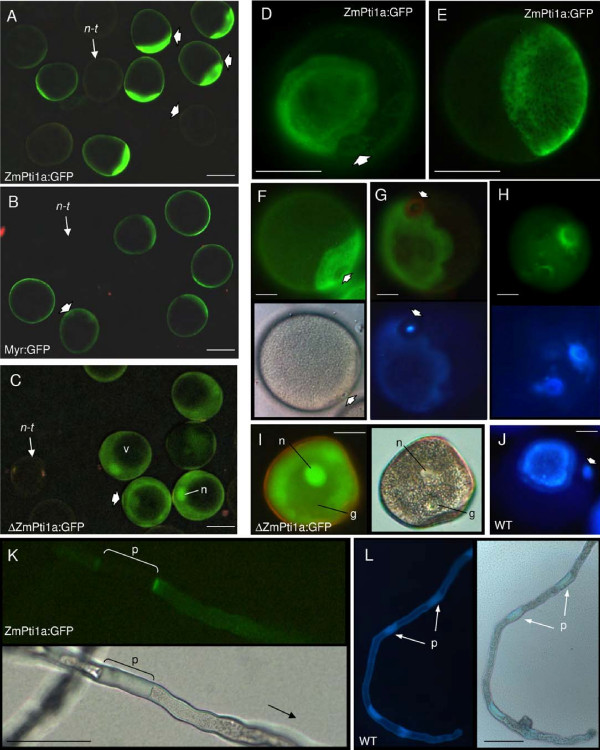
**Expression of ZmPti1a:GFP fusions in maize pollen**. Transgenic maize lines ectopically expressing wild type ZmPti1a:GFP, Myr:GFP or ΔZmPti1a:GFP (see legend Fig. 2) were generated by biolistic transformation. Immature and mature pollen of segregating plants were harvested between 8 dba and anthesis and examined by epifluorescence microscopy. non-transgenic pollen (n-t); vacuole (v); vegetative nucleus (n); generative nucleus (g), septum (s); pollen apertures are indicated by arrows. **(A) **ZmPti1a:GFP expression in segregating pollen 6 dba. **(B) **Myr:GFP expression in segregating pollen 6 dba. **(C) **ΔZmPti1a:GFP expression in segregating pollen 6 dba. **(D-F) **ZmPti1a:GFP expresed in trinucleate pollen. GFP fluorescence is visible as an annulus-ring structure adjacent to or surrounding the pollen pore. **(G, H) **GFP fluorescence (top) and decolorized aniline blue staining (bottom) of ZmPti1a:GFP expressing trinucleate pollen. **(I) **Immature binucleate pollen expressing ΔZmPti1a:GFP. GFP expression is visible in the cytoplasm and the vegetative nucleus but not in the generative cell (left); bright field image of the same pollen (right). **(J) **Trinucleate pollen of wild type line H99 stained with decolorized aniline blue and showing callose deposition as annulus-ring structure adjacent to the pore. **(K) **Tube of *in vitro *germinated ZmPti1a:GFP pollen on PGM showing GFP epifluorescence bordering a callose plug (p); black arrow: direction of pollen tube growth; bottom: bright field image. **(L) **Decolorized aniline blue staining (left) and bright field image (right) of *in vitro *germinated A188 pollen with callose plugs (p). Scale bars in panels A – E, K, L = 50 μm; panels F – J = 20 μm

To ascertain whether ZmPti1a encodes a functional protein kinase, purified bacterially expressed ZmPti1a protein fused to a His-Tag was incubated in buffer containing Mn^2+ ^and radiolabelled ATP. The *in vitro *kinase assay revealed ZmPti1a to be capable of autophosphorylation (Fig 5B ZmPti1a-His). A highly conserved lysine residue in subdomain II was shown to be necessary for kinase activity of most protein kinases (Fig. 1A). Replacement of this lysine with an asparagine (K96N) completely abolished the autophosphorylation of tomato SlPti1 [5]. When the corresponding lysine residue (K100, Fig.1A) of ZmPti1a was mutated in a similar manner (ZmPti1a-His K100N), autophosphorylation was also abolished, indicating that ZmPti1a indeed encodes a functional protein kinase (Fig. 5B) similar to SlPti1.

Tomato SlPti1 can physically interact with and be phosphorylated by SlPto on serine and threonine residues [5,25]. We hence used bacterially expressed autophosphorylation deficient GST-SlPti1(K96N) and MBP-SlPto as positive controls in our experiments (Fig. 5B). In addition, we tested the ability of tomato SlPto to phosphorylate maize ZmPti1a *in vitro*. To distinguish between autophosphorylation of ZmPti1a and cross-phosphorylation by SlPto, the phosphorylation deficient ZmPti1a-His(K100N) was used as substrate (Fig 5B). MBP-SlPto could be shown to phosphorylate maize ZmPti1a-His(K100N). We further tested if ZmPti1a-His can cross-phosphorylate tomato SlPti1. No significant phosphorylation of GST-SlPti1(K96N) could be detected in this reaction. These results indicate that maize ZmPti1a can serve as an *in vitro *substrate of tomato SlPto but is not able to cross-phosphorylate SlPti1.

### ZmPti1 serves as a substrate for kinase activities from pollen and silks

To identify upstream kinase activities which use ZmPti1a as a substrate, magnetocapture protein interaction assays were performed. Immobilized autophosphorylation deficient mutant ZmPti1a-His(K100N) was incubated with native protein extracts from pollen, silks and seedlings, respectively. After removal of unbound proteins by extensive washing, *in vitro *kinase assays were performed to detect bound kinase activities capable of phosphorylating ZmPti1a-His(K100N). ZmPti1a-His(K100N) was phosphorylated by proteins enriched from pollen and silk extracts but not from seedlings (Fig. 5C). As controls, the same pull-down experiments were performed using either autophosphorylation active ZmPTI1a-His protein or an immobilization matrix that did not contain recombinant proteins. No radioactively labeled proteins were detected in the size range of ZmPti1a-His, though silk and seedling extracts showed increased unspecific protein binding to the unloaded matrix. Cross-phosphorylation of ZmPti1a-His(K100N) by pollen and silk but not seedling protein could be verified in direct kinase activity assays without upstream pull-down purification of interacting proteins.

Auto – and/or cross-phosphorylation of the wild type ZmPti1a-His was still detectable after pull-down experiments. However, ZmPti1a-His phosphorylation was significantly weaker after incubation with proteins from pollen and silk extracts but unaltered with seedling proteins. One explanation for the reduced phosphorylation of ZmPti1a could be the presence of phosphatases or inhibitors of ZmPti1a autophosphorylation in pollen and silks extracts.

Our experiments provide evidence that pollen and silks contain kinases which bind and phosphorylate ZmPti1a *in vitro*. No such activities were detected in seedling tissue.

### ZmPti1a:GFP co-localizes with callose deposition in pollen

To study the subcellular localization of ZmPti1a in pollen *in vivo*, we generated transgenic maize lines ectopically expressing ZmPti1a:GFP, ΔZmPti1a:GFP and Myr:GFP, respectively. In each case, pollen was harvested between 8 days before anthesis (dba) and anthesis from at least four independent transgenic lines. All plants transformed with a particular construct revealed identical results.

ZmPti1a:GFP and Myr:GFP localize to the pollen plasma membrane (Fig. 6A, B). whereas ΔZmPti1a:GFP was present in the cytoplasm and the vegetative nucleus (Fig. 6C) but absent in the generative nucleus of binucleate developing pollen as well as in sperm cells of trinucleate mature pollen (Fig. 6I and data not shown). Similar localization patterns were also observed in other tissues of the respective transgenic lines (data not shown). These results are consistent with the transient localization studies in onion epidermal cells. However, ZmPTI1a-GFP was not evenly distributed over the plasma membrane but accumulated in a specific region of the pollen. This pattern was confirmed by confocal laser scanning microscopy (data not shown) and was found to be present in pollen analyzed from all plants of the four independent transgenic lines. The pollen from plants over-expressing the Myr-GFP construct showed the same pattern as seen with the ZmPTI1a-GFP construct, however to a much weaker extent with a more even distribution of the protein. This indicated that the myristoylation signal along with the additional amino acids at the N-terminus can somewhat trigger this phenomenon. However, the entire ZmPTI1a protein seems to be essential for strong protein accumulation in a specific region of the pollen. A more detailed study of the morphology of this uneven localization of ZmPTI1a-GFP revealed that ZmPTI1a-GFP accumulation is closely associated with the pollen aperture (pore). GFP fluorescence was observed below the intine surface as an annulus-ring structure which has a stronger fluorescence in the inner ring of the annulus (Fig. 6D, E). It appeared to be localized either adjacent to or surrounding the pollen pore, the former case being more predominantly observed. The position of the adjacent annulus was found to be highly conserved at a 45° angle with respect to the pollen pore.

In search for an explanation of this pattern, we investigated the possibility of ZmPti1a:GFP being associated with (1,3)-β-glucan (callose) by conducting co-localization studies with aniline blue stained transgenic pollen. ZmPti1a:GFP pollen stained for callose showed a clear co-localization of callose and GFP with the same intensity pattern, i.e. having stronger fluorescence in the inner ring of the annulus (Fig. 6G). There was no co-localization of GFP with the callose plug present in the center of the pollen aperture (Fig. 6G). Identical (1,3)-β-glucan annulus-ring structures were observed in pollen from several wild type lines proving that the ring structure is not an artifact because of ectopic ZmPti1a:GFP expression (Fig. 6J and data not shown). Some pollen showed distinct GFP localization in granules, plates and rings which also co-localized with (1,3)-β-glucan (Fig. 6H).

Transgenic ZmPti1a:GFP pollen germinated on silks as well as on PGM. In germinating transgenic pollen the fusion protein was still sequestered at the plasma membrane adjacent to the pore with only a marginal fluorescence observed in the elongating tube. However, in rare cases, stronger fluorescence signals appeared in the pollen tube bordering callose plugs (Fig. 6K). Plugs are regularly produced behind the tip as tubes elongate to isolate the cytoplasmic contents of the tube from the now empty pollen grain (Fig. 6L).

### ZmPti1a:GFP co-localizes with callose during pollen mitosis I

Various stages of pollen development were studied to further investigate the co-localization of callose and ZmPti1a:GFP.

**Figure 7:**
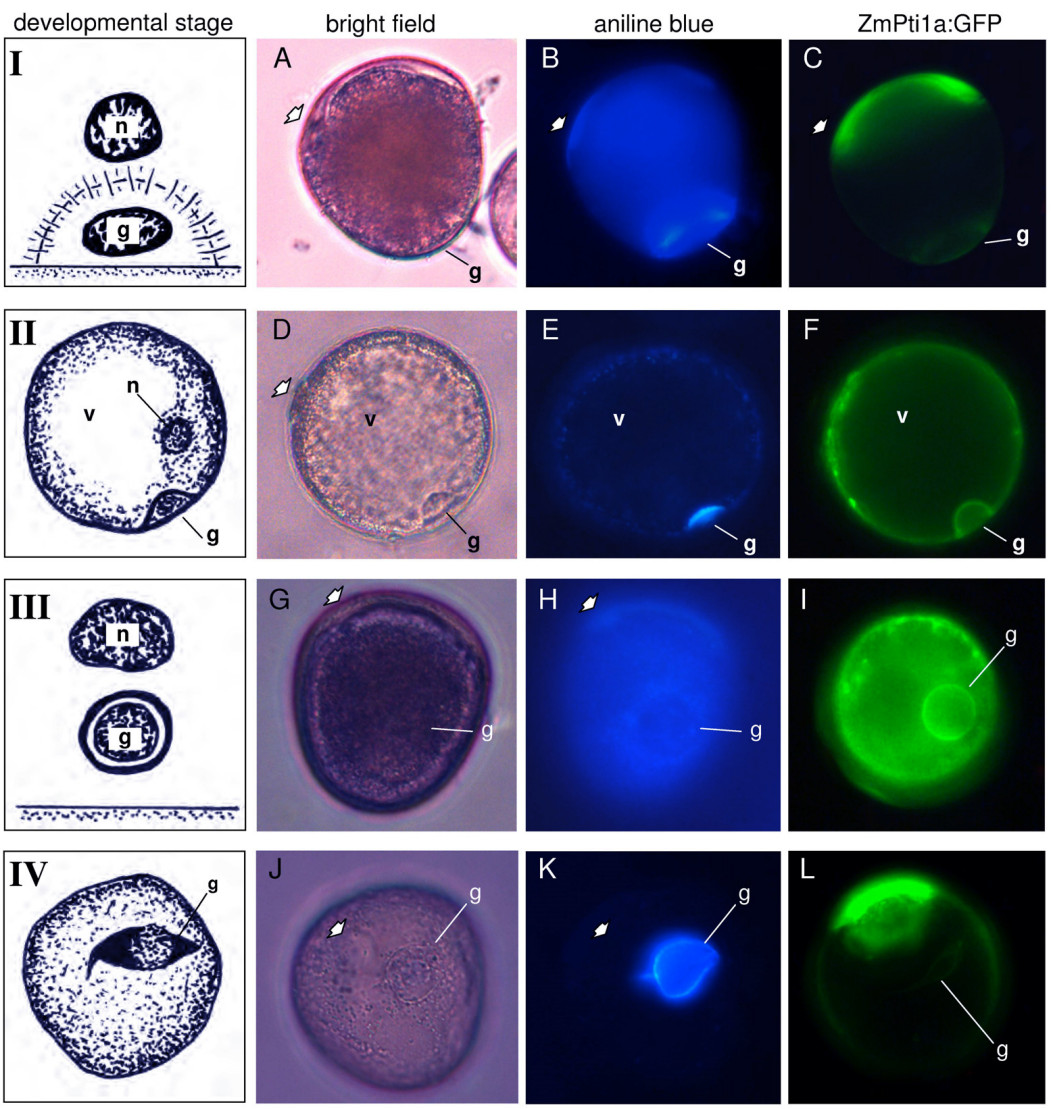
**ZmPti1a:GFP localization during pollen mitosis I**. Bright field, decolorized aniline blue staining and GFP epifluorescence of representative ZmPti1a:GFP transgenic pollen grains at four stages of pollen development. **(I) **Callose stage; **(II) **Compact callose stage; **(III) **Circular shaped prophase generative nucleus; **(IV) **Spindle shaped prophase generative nucleus. At this stage, the generative cell is still encased by callose and becomes spindle shaped. Later on, callose disappears and the generative cell immediately undergoes a second mitotic division resulting in trinucleate mature pollen. Stages I and III redrawn from [26]; stage II drawn according to [61]; stage IV redrawn from [62]; g = generative cell; n = vegetative nucleus; v = vacuole; A/B, G/H and J/K, show the same pollen grains, respectively.

The first indication of cell plate deposition in the equatorial region of the phragmoplast appeared soon after telophase during pollen mitosis I (PMI), taking the normal formation of a flattened aggregate of unit-membrane bounded vesicles. From the earliest presence of these vesicles, the equatorial zone reveals callose fluorescence. This is called the "Pre-callose stage". Thereafter, the equatorial zone continued to spread at the margins, following the out-spreading phragmoplast, until it forms a complete hemisphere. After the cell-plate formation, callose forms an arch-shaped layer which gradually reached the pollen wall, thus completely separating the generative nucleus from the vegetative nucleus (Fig. 7 stage I; A, B). During the time interval when the generative cell abuts against the wall, callose occurs at the boundary of the cell, however, only on the side adjacent to the vegetative cell. This is called the "Callose stage" (Fig. 7B; [26,27]). Following this stage, a rather compact callose stage is observed which is further defining this cell-cell boundary (Fig. 7 stage II; D, E). Soon after, the generative cell gradually changes from a lens-like to a spherical shape and moves towards the centre of the pollen grain (Fig. 7 stage III; G, H). This is the "Circular shaped prophase generative nucleus with nucleolus stage". In the "Spindle shaped prophase generative nucleus with nucleolus stage" it becomes spindle shaped (Fig. 7 stage IV; J, K) following the disappearance of callose [26,27]. In the case of maize, the generative cell immediately undergoes a second mitotic division which produces two sperm cells resulting in trinucleate mature pollen.

ZmPTI1a-GFP fusion protein was found to co-localize with (1,3)-β-glucan in association with the generative cell until the end of stage IV. When callose disappeared after the end of stage IV, neither the generative cell nor the two sperm cells during further pollen maturation were encased by ZmPti1a:GFP fluorescence. However, the fusion protein was present as the previously described annulus-ring structure adjacent to the pore. It appears that the annulus-ring structure may be associated with a similar structure from previous observations of (1,3)-β-glucan staining during pollen germination [28,29]. However, in our study this structure appeared at all studied stages of pollen development, with increasing intensity during maturation of the pollen grain.

### Knock down of *ZmPti1a *reduces male specific transmission of the transgene

To decipher the biological function of ZmPti1a during pollen development and/or germination, a targeted RNA interference (RNAi) gene silencing approach was utilized. Transgenic plants were obtained by biolistic transformation of embryogenic calli and subsequent plant regeneration using the co-transformed phosphinothrycin (PPT) resistance gene *pat *as a selection marker. Fifteen out of 20 regenerated primary T_0 _plants carried the RNAi construct. Southern analyses showed that these plants corresponded to 9 independent clonal lines. Each line contained multi-copy integrations of the RNAi construct. Southern studies comprising of T_0 _plants and segregating individuals of three subsequent generations indicated that the line specific integration patterns of the transgene were inherited as a single genomic locus (data not shown). Northern analyses with leaf and pollen RNA using a transgene-specific probe confirmed expression of the RNAi construct in all lines (data not shown).

The T_0 _plants were reciprocally crossed with the wild type line A188. It was observed in initial experiments that the RNAi transgene was not transmitted in the expected Mendelian ratio. In order to score for competitive ability of RNAi transgenic pollen, 234 offspring plants were assayed for PPT-resistance. Co-segregation of RNAi and PPT transgenes was confirmed by Southern analyses of 77 PPT-resistant T_1 _individuals.

**Figure 8:**
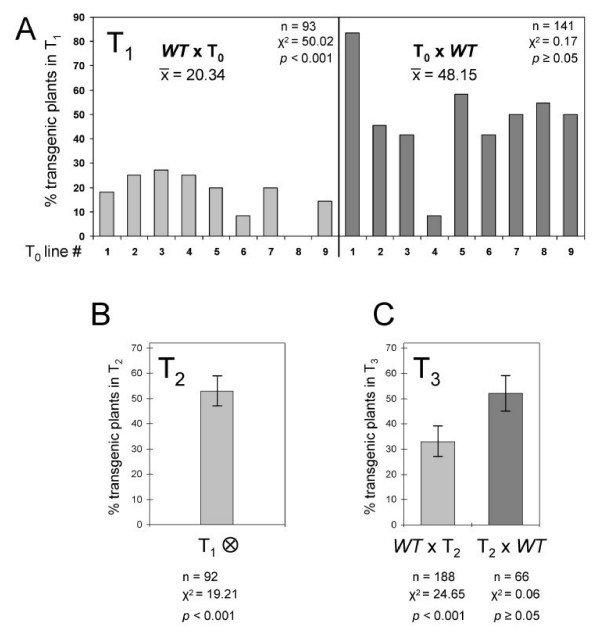
**Genetic segregation analyses of transgenic *ZmPti1a*-RNAi plants**. **(A) **T_0 _plants expressing a *ZmPti1a *directed RNAi construct were crossed reciprocally to wild type A188. Transmission of the RNAi transgene was scored in T_1 _siblings of each line as a percentage of 'transgenic PPT-resistant plants' to 'PPT-sensitive plants'. The progeny of wild type plants pollinated with pollen of transgenic plants (left) showed a significant difference from a 1:1 segregation ratio with a lower than expected marker transmission (Probability value based on χ^2 ^test p < 0.001; n = number of individuals analyzed). **(B) **T_1 _transgenics were selfed and transmission of the transgene was scored in the T_2 _progeny as described. Plants showed a significant difference from the expected 3:1 segregation with a lower than expected marker transmission of only 53%. **(C) **Eight different heterozygous T_2 _transgenic plants were reciprocally crossed to wild type A188. Marker transmission was scored as described. Only the T_3 _progeny of wild type plants pollinated with pollen of transgenic plants showed a difference from a 1:1 segregation ratio. Marker transmission was significantly lowered to 33%.

When transgenic plants were pollinated with wild type pollen, 48% of the T_1 _progeny showed PPT-resistence and carried the RNAi transgene, respectively (Fig. 8A; T_0 _× WT). Such an apparent 1:1 segregation is expected for a single locus inheritance. When wild type plants were pollinated with pollen of transgenic T_0 _lines, only 20% of the T_1 _progeny was transgenic (Fig. 8A T_0 _× WT). Since abnormal segregation was only observed with transgenic pollen, the result implied that the transgene was transmitted normally through the female but at a lower frequency through the male.

**Figure 9:**
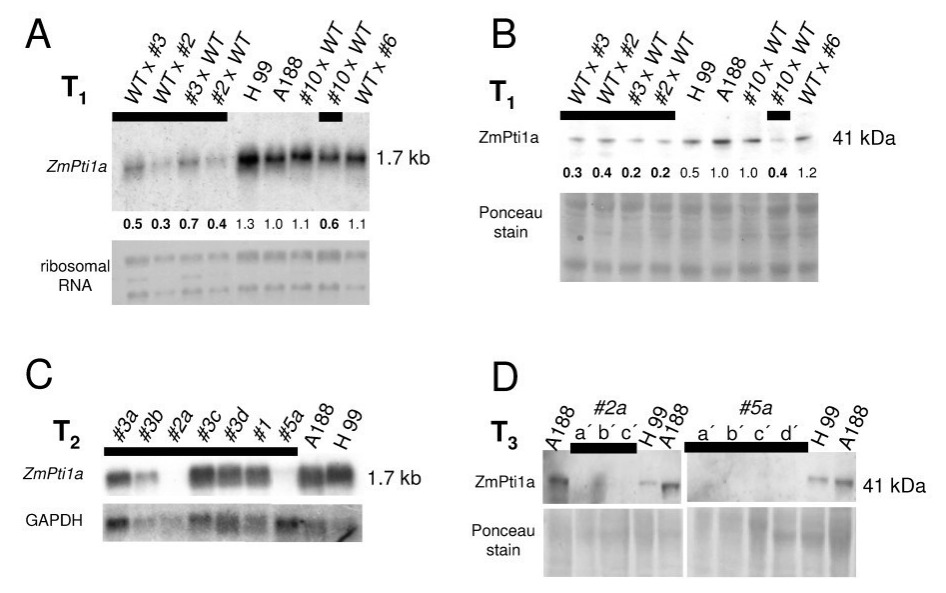
***ZmPti1a *mRNA and protein expression in *ZmPti1a*-RNAi transgenic plants**. **(A) **Northern and **(B) **Western analysis of *ZmPti1a *expression in pollen of wild type (H99, A188), T_1 _heterozygous transgenic (black bar), and PPT-resistant plants lacking the RNAi construct (#10 × WT; WT × #6). *ZmPti1a *mRNA and protein levels were quantified densitometrically and mRNA and protein levels in A188 were arbitrarily set to 1, respectively. All other values were calculated as multiples of that. Values of *ZmPti1a-*RNAi transgenic individuals are indicated in bold. Methylene blue staining of ribosomal RNA and Ponceau staining of proteins are given as a control for loading **(C) **Northern analysis of *ZmPti1a *expression in pollen of T_2 _descendants from selfed heterozygous T_1 _plants. Pollen of plants #2a and #5a contain no detectable levels of *ZmPti1 *mRNA. Expression of GAPDH was used as a loading control **(D) **Western analysis of ZmPTI1a protein expression in pollen of T_3 _siblings from descendants of plants #2a and #5a. ZmPTI1 protein was decreased to undetectable levels in all individuals analyzed. Numbers refer to transgenic lines; lower case letters indicate siblings of the same line.

To confirm the RNAi effect on *ZmPti1a *expression, T_1 _plants were subjected to Northern and Western analysis. *ZmPti1a *transcript and protein levels were reduced to approximately 50% in all analyzed transgenic plants (Fig 9A, B). This finding is in agreement with the heterozygous RNAi genotype of the transgenic T_1 _siblings. Accordingly, non-transgenic individuals of the segregating T_1 _lines showed normal *ZmPti1a *expression (Fig. 9A, B; #10 × WT, WT × #6).

In order to further score transgene transmission, T_1 _plants were selfed and their progeny (T_2_) was analyzed for PPT-resistence. T_2 _plants showed apparent 1:1 segregation (Fig. 8B) which differed clearly from the expected 3:1 Mendelian segregation and further supported an impaired competitive ability of transgenic RNAi pollen. Hence, reciprocal crosses between transgenic and wild type plants were repeated with 8 different heterozygous transgenic T_2 _plants. Segregation analysis of 254 T_3 _siblings showed only 33% transgene transmission for the cross WT × T_2 _but 52% for the cross T2 × WT. This result confirmed a male specific reduction of transgene transmission (Fig. 8C).

To check for the existence of homozygous RNAi lines, T_2 _plants were screened by Northern analysis using pollen RNA. Several individuals could be identified in which *ZmPti1a *expression was reduced to undetectable levels (Fig. 8C; #2 and #5; and data not shown). After selfing these plants, the corresponding T_3 _progeny did not segregate and ZmPTI1a protein was decreased to undetectable levels in pollen (Fig. 9D), indicating the successful establishment of homozygous RNAi lines with a silenced *ZmPti1a *gene. Homozygous RNAi plants did not show any visible mutant phenotype. Since homozygous RNAi lines could be generated and propagated, knock down of *ZmPti1a *did not result in male sterility. Accordingly, RNAi pollen was able to germinate and did not show reduced pollen tube growth rates *in vitro *(data not shown). Because ZmPti1a was found to be associated with callose deposition, pollen from heterozygous and homozygous T_2 _RNAi plants was stained for (1,3)-β-glucan. Microscopic examination did not reveal significantly altered callose patterns. Relative (1,3)-β-glucan amounts were assayed in pollen of ca. 100 T_0 _to T_3 _plants and of wild type lines A188 and H99 modified according to Köhle *et al. *[30]. However, the analysis did not result in statistically significant differences between wild type and homo- or heterozygotic RNAi pollen, mainly due to a generally high variation in callose content. Taken together, knock down of *ZmPti1a *expression did not result in a visible pollen defect. However, crossing studies indicated that *ZmPti1a *expression provides a significant competitive advantage to the male gametophyte and demonstrated *ZmPti1a *to be an important component for maize reproduction.

## Discussion

ZmPti1a, -b, -c and -d are the first ZmPti1-like kinases in a monocotyledonous species to be characterized at the molecular level. Although ZmPti1 kinases from maize do not display high similarity at the nucleotide sequence level, the translated protein sequences are well conserved and the kinase domains maintain all functionally important residues. Accordingly, *ZmPti1a *was proven to encode a functional kinase capable of autophosphorylation and to be an *in vitro *target of kinase activities present in pollen and silk tissue. The presence of several Pti1-like kinases in maize is consistent with the observations of *Pti1*-genes belonging to a gene family in tomato [4] and three *Pti1*-related genes identified in *Glycine max *[8]. Phylogenetic analysis revealed a wide diversity of Pti1 homologues in the plant kingdom, appearing to segregate into three sub-families mainly based on different N-terminal sequences of the kinases. These sequence regions were not only specific to the maize Pti1-kinases but were also highly conserved in putative Pti1 kinases of other monocotyledonous and dicotyledonous species. This gives rise to the conclusion that conserved N-termini in different Pti1 sub-families are evolutionary conserved indicating the functional relevance of this protein domain for proper biological function. Accordingly, studies with GFP fusions suggest that the variable N-termini are critical for different targeting properties of the four maize Pti1 kinases.

ZmPti1a contains an N-terminal putative myristoylation motif essential for plasma membrane targeting. N-myristoylation is known to confer reversible membrane association by enhancement of hydrophobic membrane-protein interactions. Such membrane associations are known to be further stabilized by reversible S-palmitoylation of Cys residues on positions 3 to 5 [31]. N-terminal Gly2 and S-palmitoylation at Cys4 and Cys5 of OsCPK2 has been shown to occur *in vivo *in maize leaf protoplasts with both acylations contributing to full membrane association [32]. ZmPti1a contains Cys residues in position 3, 6 and 7. Hence, it is likely that these motifs are subjects of fatty acylation and are necessary for proper plasma membrane targeting. Indeed, site-directed mutagenesis of the corresponding residues partially abolished plasma membrane targeting of ZmPti1a:GFP.

Interestingly, dual S-acylation was shown to influence protein compartmentalization to specialized plasmalemmal microdomains [33-35]. It is tempting to speculate that microdomain association is also responsible for the unequal distribution of ZmPti1a in the pollen plasma membrane.

N-terminal myristoylation is not only required for membrane targeting but could also participate in intra- or intermolecular interactions with proteins [31,33]. For instance, intermolecular myristate-protein interactions were shown to facilitate interaction between calmodulin and several proteins [36-38]. Therefore, ZmPti1a plasma membrane targeting could also be achieved indirectly by interacting proteins. Evidence for ZmPti1a interacting proteins is provided by the magnetocapture interaction assays which suggested the existence of ZmPti1a phosphorylating kinases that bind and phosphorylate ZmPti1a *in vitro*.

Mutation of single amino acids which are putatively relevant for myristoylation or acylation results in partial nuclear location of ZmPti1a. Partial nuclear targeting was also observed for ZmPti1b and SlPti1. In both proteins Gly2 is naturally substituted by Ser2. Chehab *et al. *[39] demonstrated that the N-terminally myristoylated calcium-dependent protein kinase (CDPK) McCPK1 from *Mesembrynathemum chrystallinum *undergoes a reversible change in subcellular localization from the plasma membrane to the nucleus, endoplasmic reticulum, and actin microfilaments in response to reduction in humidity. Proteins of the CDPK family also contain variable N-termini and exhibit diverse subcellular localization patterns which reflect their participation in a variety of signaling pathways including HR [40,41]. Reversible translocation and activity-dependent localization was also shown for other myristoylated proteins e.g. for the calcium-myristoyl switch proteins VILIP1 and VILIP3 in neurons and the flagellar calcium-binding protein FCaBP in *Trypanosoma cruzi *[42,43]. Thus, it will be of profound interest to investigate if ZmPti1a can respond to specific signals by changing its subcellular localization and if putative downstream targets of ZmPti1a signaling reside inside the vegetative nucleus of pollen.

ZmPti1c and ZmPti1d did not contain myristoylation signals but conserved N-terminal pairs of arginine residues. GFP fusions of these sub-group III Pti1 proteins showed neither membrane nor nuclear targeting. This is in agreement with studies showing that arginine residues favor cytoplasmic displacement and reduce protein-lipid or protein-protein interactions [44].

ZmPti1a co-localizes with plasma membrane regions responsible for callose deposition during pollen development and tube growth. Callose is a (1,3)-β-glucan with some 1,6 branches which not only is the major component of the pollen grain and pollen-tube wall but also plays a key role in pollen viability. Callose is deposited in the inner wall layer behind the growing pollen tube tip as well as in regularly formed plugs inside the pollen-tube. Callose is not specific to the male gametophyte but widely distributed among various cells and tissues. In sporophytic tissue, synthesis of callose is evidently achieved *via *a plasma membrane bound multi-subunit enzyme that is usually activated temporally by specific signals such as wounding, infection or other stress. Twelve callose synthase (CalS) isoenzymes have been identified in *Arabidopis *and each may be tissue-specific and/or regulated under different physiological conditions [45]. Putative regulatory subunits of the CalS complex are ROP1 GTPase, UGT1, annexin-like proteins (ANN) and sucrose synthase [45]. However, subunit composition and regulation of CalS complexes is far from being understood. Remarkably, Arthur *et al. *[18] showed that ROP2, one of 9 known ROP GTPases from maize, also provides a competitive advantage to the male gametophyte. Heterozygous *rop2::Mu *mutants show a reduced transgene transmission by pollen similarly to what was observed with *ZmPti1a *RNAi plants. Although there is no experimental evidence yet, a biological link between ROP GTPases, CalS complexes and ZmPti1a may be speculated for pollen.

In accordance with the presence of multiple CalS complexes in plants, callose deposition is not only known to be involved in pollen development, pollen-tube growth and self-incompatibility but also in cytokinesis and pathogen defence. For instance, hypersensitive cell death induced in plants thwarts further advance of the pathogen is seen to correlate with callose deposition at and around the site of HR. This strengthens the cell wall to attenuate pathogen invasion [46]. In tomato, SlPto and SlPti1 were shown to be involved in gene-for-gene resistance against *Pseudomonas syringae *pv *tomato *strains. Based on protein similarity and subcellular localization ZmPti1b was identified as a likely functional ortholog of SlPti1 in maize. A putative function of ZmPti1b in pathogene defense was supported by the observed induction of ZmPti1b transcript in *Fusarium *spec. infected kernels.

The diversity of callose function exemplified increasing evidence that plant responses such as male fertility, self-incompatibility, pathogenesis, symbiosis and wounding are based on highly related molecules and have evolutionary conserved signalling mechanisms [1-3]. Our analyses of Pti1 kinases from maize support this model and suggested Pti kinases as conserved components of these different but related signal cascades in plants. Current studies are aimed at further elucidating the function of ZmPti1 genes in maize and in the model plant *Arabidopsis thaliana*.

## Conclusion

In this study we cloned and characterized four novel Pti1 kinases from maize. Expression patterns and subcellular targeting of the proteins were investigated. ZmPti1a encodes a functional serine/threonine kinase specific to pollen and is associated with plasma membrane regions responsible for callose deposition. ZmPti1a expression provides a competitive advantage to pollen. Thus its activity seems to be important for the proper biological function of pollen. In contrast, ZmPti1b is likely to be involved in maize pathogen responses. Considering the fact that pollen-sporophyte interaction and pathogen induced HR show certain similarities e.g. callose deposition and Ca^2+ ^influx, members of the Pti1 kinase family are hypothesized to act as general components in evolutionary conserved signalling processes during different developmental programs and in different tissue types.

## Methods

### Plant material and cultivation conditions

The maize lines used in this study were inbred lines LC (a color converted W22 line), A188, H99, and a flavonol-deficient conditionally male-sterile line "*white pollen*" (genotype: *whp/whp*, *c2/c2*; [22]) and its corresponding fertile wild type line (*whp/whp*, *C2/C2 *or *whp/whp*, *C2/c2*). Plants were grown in a green house with 16 h of light at 24°C and a relative humidity of 55% to 95%. Controlled pollination was achieved by the use of standard breeding procedures.

For expression analyses different maize tissue types were harvested as described previously [47].

Maize pollen used for microscopic studies was collected at different stages of development. Early samples representing a mixture of unicellular microspores and bicellular immature pollen were harvested from anthers 6 to 8 dba before their emergence from the flag leaf. A mixture of immature and mature pollen was harvested between 2 dba and anthesis. Mature pollen was harvested at the pollen shading stage.

### *In vitro *germination of pollen

Fresh pollen was harvested between 11 and 12 a.m. and was germinated according to [48]. 30–50 mg pollen was germinated in 1 to 2 ml of liquid pollen germination medium (1 × PGM) in 35 mm petri dishes. Germination was monitored by light microscopy. For RNA or protein extraction germinating pollen was harvested by centrifugation, frozen in liquid nitrogen and stored at -70°C until use.

### Aniline blue staining of maize pollen

Maize pollen was stained with 1% Aniline blue (Merck, Darmstadt) which was prepared in 0.07 M Na_2_HPO_4 _pH 8.5 (modified according to [49]). The samples were excited with UV light produced by an HBO 50 W/Ac lamp using a filter set (excitation BP 365/12, FT395 chromatic beam splitter and emission LP 397 nm). For documentation of the results an Olympus C4040 zoom digital camera was used. An AxioCam MRc5 camera (Zeiss, Germany) and software packages AxioVS40AC V 4.1.1.0 and ImageJ 1.34n [50] were used for digital imaging.

### *ZmPti1 *cDNA isolation

To obtain a full-length *ZmPti1 *cDNA clone, a lambda cDNA library was generated from mRNA of 10 min *in vitro *germinated fertile *whp *pollen (*whp/whp*, *C2/C2 *or *whp/whp*, *C2/c2*) using the *SMART*™*cDNA Library construction kit *(BD Biosciences). Library construction was performed as described in the manufacturer's manual. Amplified cDNAs were ligated into *Sfi*I restricted λ *TriplEx2*™ vector and packed with Gigapack^® ^III Gold Packaging Extract (Stratagene, Heidelberg, Germany). Plaque screening was achieved using standard procedures using a Digoxigenin-labeled partial *ZmPti1a*-cDNA fragment of 217 bp as a probe. cDNA inserts of positive clones were converted into plasmids by *Cre/lox *mediated recombination into BM25.8 *E. coli *cells according to the *SMART*™*cDNA Library construction *manual (BD Biosciences).

3'-UTRs of *ZmPti1b*, -*c *and -*d *were amplified by PCR using gene specific primer pairs; SP2 (5'-atgcgcgggcgactaaccctggagaacatg-3') and SP3 (5'-ccgagcctggaggcattctgttcaga-3') for ZmPti1b; SP7(5'-cgcaaccaccggcagccactactgacgcta-3') and SP8 (5'-taataaggtggtcacgaccgctg-3') for ZmPti1c; SP12 (5'-ctgcaccaaccaccgaagagccagctcca-3') and SP13 (5'-aacttcgaaccaacttcatataccatt-3') for ZmPti1d; and λ-ZAP^® ^II cDNA-libraries prepared from kernels and seedlings of line LC, respectively [51]. Corresponding ORF sequences were amplified by RT-PCR from mRNA of developing kernels from the line A188. The primers used were: SP4 (5'-acagatctatgtcgtgctttgcgtgc-3') and SP5 (5'-gcagatctgacccagcatgttctcc-3') for *ZmPti1b*; SP9 (5'-gtagatctatgcgccggtggttttgc-3') and SP10 (5'-agagatctgcatctgacggtgctgt-3') for *ZmPti1c *and SP14 (5'-atagatctatgaggcggtggttatgt-3') and SP15 (5'-agagatctctcccaggttgtgg-3') for *ZmPti1d*.

### *ZmPti1a *gene isolation

A λ *Fix*^® ^*II *genomic library of the inbred line LC [51] was plated and screened following standard protocols by using a *ZmPti1a-3'UTR *probe generated by PCR with primers PRMMH53 (5'-tgtgatttctcatcgctgcg-3') and PRMMH52 (5'-cggaagccaacgctgcattttcgc-3'). λ-DNA was isolated from phages of interest according to [52]. Southern analysis with different *ZmPti1 *cDNA probes indicated that one of the phage clones contained the complete *ZmPti1a *gene. For sequencing, a 1.4 kb *Pst*I fragment of the *ZmPti1a *ORF was cloned into pBluescript KS^-^. Three additional fragments spanning the entire transcribed region of the gene were amplified with primer pairs PRMMH52 and PRMM55 (5'-ggagtcgcatatgggatgcttttcatgctg-3'), PRMMH53 and T3 (5'-attaaccctcactaaag-3') and PRMMH85 (5'-ccgcgaggcattctgaaatcg-3') and PRMMH70 (5'-gtggtactagcaagcatgataa-3'), respectively. All three fragments were cloned into pCR2.1-TOPO (Invitrogen, Karlsruhe, Germany) and sequenced. 3.0 kb of the *ZmPti1a *promoter were amplified from a 4.1 kb *Pst*I fragment of the isolated λ-phage by inverse PCR according to [53] with the primers PRMMH62 (5'-atggccgactccctaacct-3') and PRMMH75 (5'-acaaaaagggcttcctgtgc-3').

### Sequence analysis

Sequencing and bioinformatics were performed as described previously [47]. The myristoylation pattern was identified with the *Plant Specific Myristoylation Predictor *software [54].

### Northern blot analysis

Total RNA from maize tissue was isolated and blotted as described previously [47]. As a control for equal RNA loading, blots were stained with 0.05% methylene blue; 0.3 M sodium acetate (pH 5.0) for ten minutes and destained in deionized water. ^32^P-dCTP (3000 Ci/mmol)-labeled probes were generated with the *Prime It*^® ^*II *DNA labeling kit (Stratagene, Heidelberg, Germany) and hybridized under stringent conditions [47].

### Cloning of a *ZmPti1a*-RNAi construct

Expression of the *ZmPti1a*-RNAi transcript was performed under the control of the ubiquitin-1 promoter (*Ubi-1*) from rice and the terminator of the nopalin synthase gene (*nos*) in the pUbi.cas plasmid [55]. An *Eco*RI PCR product (*c2i*) of the *c2 *gene [56] which contained 16 bp of the 3' border of exon 1, the entire intron 1, and 39 bp of the 5' border of exon 2 was cloned into the *Eco*RI site of pBluescriptKS^- ^(*pBS*; Stratagene, Heidelberg, Germany). Two fragments (RNAi-5' and RNAi-3') of the 5'-region of the *ZmPti1a *ORF were amplified in reverse complement orientation to each other with the primer pairs PRMMH65 (5'-tagggaggtcgacatgggatgcttttcatgctgc-3') and PRMMH66 (5'-ggagtcaggatccttcactgcagatttcgtccc-3') or PRMMH67 (5'-tagggagggtaccatgggatgcttttcatgctgc-3') and PRMMH68 (5'-ggagtcaagcttcttcactgcagatttcgtccc-3'), respectively. Both primer pairs amplified appropriate restriction sites at the ends of the fragments. Products were cloned into pCR2.1-TOPO plasmids. RNAi-3' was digested with *Kpn*I and *Hin*dIII and was inserted 3' behind *c2i *into the *Kpn*I/*Hin*dIII site of *pBS*. A *Kpn*I/*Asp*718 restriction fragment of this clone containing both *c2i *and PTGS-3' was integrated in the *Kpn*I/*Asp*718 site of Ubi.cas between *Ubi-1 *and *nos*. Subsequently, the RNAi-5' fragment was excised from the pCR2.1-TOPO with *Bam*HI and ligated into the *Bam*HI site of the *ZmPti1a*-RNAi construct.

### Cloning of GFP fusion constructs

A modified GFP expression construct (pUbi:GFP) was cloned based on the pMon30049 vector [57]. The coding sequence of GFP was amplified with primers GFP-for (5'-tacggatccaggagcaaccatgggc-3') and GFP-rev (5'-tgcgaattctcagccatgcgtgatcccagc-3') with pMon30049 as template. Primer GFP-rev shortened the C-terminus of GFP by six amino acids [MDELYK], introducing a new stop codon and an *Eco*RI site. The coding sequence of GFP was excised from pMon30049 with *Bam*HI/*Eco*RI and was replaced with the *Bam*HI/*Eco*RI restricted GFP fragment. The 1.5 kb *CaMV35S *promoter of pMon30049 was excised with *Hin*dIII/*Bam*HI and replaced by the 1.5 kb *Ubi-1 *promoter that was cut with *Hin*dIII and *Bam*HI from pUbi.cas.

The coding sequence of *ZmPti1a *was amplified with primers JK3 (5'-tactggatccatgggatgcttttcatgctg-3') and JK4 (5'-tcctggatccagtccggatcgctcggcag-3') using *ZmPti1 *cDNA as template. Both primers amplified *Bam*HI sites at the N – and C-termini of the protein. The PCR product was ligated in-frame into the *Bam*HI linearized pUbi:GFP leading to the ZmPti1a:GFP construct. In order to remove N-terminal 20 amino acids of ZmPti1a, Δ ZmPti1a:GFP was cloned similarly, with the exception that the primers JK9 (5'-tactggatccatggacgatccctatgttcctatc-3') and JK4 were used for amplification. GFP fusion constructs of ZmPti1b, -c and -d were cloned similarly by using specific primer pairs which generated *Bam*HI compatible *Bgl*II restriction sites.

Myr:GFP was cloned by *in vitro *annealing of oligonucleotides Myr-A (5'-P-gatccatgggatgcttttcatgctgctgtgtggcagatgacgacaacgttggcaggaggaagaagcat-3') and Myr-B (5'-P-gatcatgcttcttcctcctgccaacgttgtcgtcatctgccacacagcagcatgaaaagcatcccatg-3') and ligation of the resulting double stranded DNA into *Bam*HI linearized pUbi:GFP. ZmPti1a:GFP mutant constructs were generated by site-directed mutagenesis using primer JK3 in combination with one of the following primers: D1 (5'-gagggatccatggcatgcttttcatgctgc-3'), D2 (5'-gagggatccatgggagccttttcatgctgc-3'), D3 (5'-gagggatccatgggagccttttcagcctgctg-3'), D4 (5'-gagggatccatgggatgcttttcagccgcctgtgtgg-3') and D5 (5'-gagggatccatggcagccttttcatgctgc-3'). PCR fragments were cloned into pCR2.1-TOPO, subcloned into *Bam*HI linearized pUbi:GFP and sequenced. The SlPti1(K96N):GFP fusion was cloned accordingly with primers JK10 (5'-tactggatccatggcacacaattcagcaggcaac-3') and JK11(5'-tcctggatcccttggtacaggtcgaggcaacag-3') and with GST-SlPti(K96N) [5] as template. GFP fusions of ZmPti1b and c were cloned in the same way.

### Expression and purification of fusion proteins

For protein expression in bacteria, the ZmPti1a kinase and its mutagenized autophosphorylation-deficient form ZmPti1a(K100N) were fused in frame with 6× histidine tags (His) at their C-terminal ends: The ORF of *ZmPti1a *was amplified with PRMMH55 (5'-ggagtcgcatatgggatgcttttcatgctg-3') and PRMMH64 (5'-gcgatggcggccgccagtccggatcgctcggcagc-3'), digested with *Nde*I and *Not*I and inserted in the corresponding sites of linearized pET30a plasmid (Novagen/Merck Biosciences, Germany). To obtain the ZmPti1a-His(K100N) construct, the K100N mutation was introduced by amplification of the 5' end of *ZmPti1a *with primers PRMMH55 and PRMMH84 (5'-ctggagtcaagcttgttcactgcagatttcgtccc-3'). A *Nde*I/*Hin*dIII fragment was cut from the ZmPti1a-His construct and was replaced by the *Nde*I/*Hin*dIII digested ZmPti1a(K100N) PCR product.

Plasmids ZmPti1a-His, ZmPti1a-His(K100N), MBP-SlPto [25], and GST-SlPti1(K96N) [5] were transformed into *E. coli *Rosetta(DE3) (Novagen/Merck Biosciences, Schwalbach, Germany) and expression of the fusion proteins was induced by adding isopropyl β-thiogalactoside to a final concentration of 1 mM and incubation for 3 h at 28°C. Batch purification of the recombinant proteins under denaturing conditions was achieved by binding to Ni-NTA agarose resin (Qiagen, Hilden, Germany). 0.5 g bacteria were harvested in 2.5 ml lysis buffer (8 M urea, 100 mM NaH_2_PO_4_, 10 mM β-mercaptoethanol, and 10 mM Tris-HCl, pH 8.0). The solution was cleared by centrifugation at 16,000 × *g *for 20 min at 4°C and the supernatant was incubated with 150 μl of Ni-NTA agarose resin for 1.5 h at 4°C. The resin was collected by centrifugation and was washed four times with washing buffer (8 M urea, 100 mM NaH_2_PO_4_, and 10 mM Tris-HCl, pH 6.3). The fusion protein was eluted with washing buffer plus 100 mM EDTA. 350 mg recombinant protein was used by Biogenes (Berlin, Germany) for rabbit immunization to generate a ZmPti1a specific polyclonal antibody. Specificity and working concentrations of the polyclonal Anti-ZmPti1a antibody serum was determined empirically with recombinant ZmPti1a-His.

For kinase assays, recombinant proteins were purified under native conditions: Bacteria expressing ZmPti1a-His and ZmPti1a-His(K100N) were harvested in His-lysis buffer (300 mM NaCl, 10 mM imidazole, 10 mM β-mercaptoethanol, 1 mM Phenylmethylsulphonylfluoride (PMSF), 0.05% (v/v) Tween^® ^20, 200 μg lysozyme, 50 mM NaH_2_PO_4_, pH 8.0), incubated on ice for 30 min and sonicated. Solubilized protein was cleared by centrifugation and incubated with Ni-NTA magnetic beads (Qiagen, Hilden, Germany) for 1 h at 4°C. Beads were separated by magnetic force and were washed thrice with washing buffer (300 mM NaCl, 20 mM imidazole, 10 mM β-mercaptoethanol, 1 mM PMSF, 0.05% (v/v) Tween^® ^20, 50 mM NaH_2_PO_4_, pH 8.0).

Induction of MBP-SlPto expression and purification of recombinant protein by binding to *amylose resin *(New England Biolabs, Frankfurt, Germany) was performed according to [58] with modifications. Cells were lysed for 30 min at 4°C in MBP-buffer (200 mM NaCl, 1 mM EDTA, 10 mM β-mercaptoethanol, 1 mM PMSF, 200 μg lysozyme, 20 mM Tris-HCl, pH 7.4) and sonicated. The cleared lysate was incubated for 1 h at 4°C with *amylose resin *and the resin was washed thrice with MBP-buffer.

Purification of recombinant GST-SlPti1(K96N) was done as described for MBP-SlPto with the exception that GST-buffer (137 mM NaCl, 2.7 mM β-mercaptoethanol, 1 mM PMSF 4.3 mM Na_2_HPO_4_, pH 7.3, 1.47 mM KH_2_PO_4_) and *GST-Bind-Resin *(Novagen/Merck Biosciences, Schwalbach, Germany) were used.

### Plant protein extraction and immunoblot analysis

Frozen tissues were ground in a MM301 ball mill (Retsch, Haan, Germany) and proteins were solubilized for 1 h at 4°C in 1 volume extraction buffer (100 mM NaCl, 5 mM EDTA, 10 mM β-mercaptoethanol, 2 mM PMSF, 1% (v/v) Triton^® ^100, 50 mM Tris-HCl, pH 7.4). Protein extract was cleared by centrifugation (10 min, 4°C, 10,000 × *g*) and the protein concentration in the supernatant was determined using Bradford reagent. For protein extraction from pollen the volume of extraction buffer was increased and 2.5 U/ml Benzonase^® ^Nuclease (Novagen/Merck Biosciences) were added to the buffer. Protein extracts of germinating pollen were concentrated on Centricon 10 (Amicon, Bevely, USA) spin columns.

Proteins were fractionated by SDS-PAGE with NuPAGE^® ^Bis-Tris gels (Invitrogen, Karlsruhe, Germany) according to the supplier's manual and transferred onto ECL-Nitrocellulose membranes (Amersham Biosciences, Freiburg) by electroblotting. Immunodetection was performed using standard ECL system procedures (Amersham Biosciences, Freiburg).

### Kinase assay

Kinase assays were performed with recombinant proteins bound to resins or magnetic beads according to [58] with modifications. Proteins were incubated in kinase assay buffer (10 mM β-mercaptoethanol, 10 mM MnCl_2_, 20 μM ATP, 10 μCi γ-[^32^P]-ATP (3000 Ci/mmol), 50 mM Tris-HCl, pH 7.0) for 20 min at 25°C and washed twice in 300 mM NaCl, 10 mM β-mercaptoethanol, 0.1% Tween^® ^20, 50 mM Tris-HCl, pH 7.0). Proteins were eluted with SDS-containing loading buffer at 85°C for 5 min and separated by SDS-PAGE. Gels were washed twice in running buffer, dried (Gel Drying System 583, Bio-Rad Laboratories, München, Germany) and subjected to autoradiography.

For magnetocapture protein interaction assays, purified recombinant protein bound to magnetic beads was washed and equilibrated in interaction buffer (300 mM NaCl, 20 mM imidazol, 10 mM mercaptoethanol, 1 mM PMSF, 0.05% (v/v) Tween^® ^20, 50 mM NaH_2_PO_4_, pH 8.0) and incubated with 40 μg native plant protein at 4°C for 1 h under agitation. After extensive washing of bound protein with interaction buffer, remaining protein was used in the kinase activitiy assays as aforementioned.

### Transient transformation of plant tissue

Pollen was harvested at anthesis and was germinated on 1 × PGM supplemented with 0.3% (w/v) agar (Duchefa, Haarlem, The Netherlands). Bulbs of *Allium fistulosum *L. or *Allium cepa *L. were sliced into quarters, innermost peels were removed and the inner sides of the quarters were used for biolistic transformation.

3 μg of plasmid DNA were precipitated on 1.25 mg/25 μl gold particles (0.3 – 3.0 μm Chempur, Karlsruhe, Germany), by adding 10 μL 0.1 M spermidine and 25 μL 2.5 M CaCl_2_. The precipitated DNA was washed once with ethanol and resuspended in 65 μL ethanol. 10 μl of this solution was spread on the macrocarrier for each bombardment. Biolistic transformation was performed with a particle gun, PDS-1000/He (Bio-Rad, Munich, Germany) as described previously [59]. A partial vacuum of 98 kPa and a gas pressure of either 10.7 MPa (1550 psi) for pollen or 7.6 MPa (1100 psi) for onion epidermis was used for bombardment. Fluorescence microscopy of GFP expressing pollen or onion cells was carried out after over night incubation at 20°C using a fluorescence microscope (Axioskop, Zeiss, Germany) equipped with a HBO 50 UV lamp using a filter set (excitation BP450-490 nm, FT 510 chromatic beam splitter, and emission LP 520 nm). Digital image capturing was performed as described for aniline blue staining.

### Regeneration of transgenic lines

Transgenic maize lines were regenerated from biolistically transformed embryogenic calli of a cross between A188 × H99 as described previously [59]. For selection of transgenic lines a *35S:pat *construct expressing the phosphinothricin acetyltransferase was co-transformed. *pat *gene expression in segregating seedlings was monitored by spraying with aqueous solution of 250 mg/l phosphinothricin and 0.1% (v/v) Tween^® ^20.

## Authors' contributions

MMH, JK and SP carried out the cloning of ZmPti1 sequences and plasmid constructs and performed expression analyses. MMH and JK performed recombinant protein expression and kinase activity studies. SP and JK carried out transient localization studies. MMH, SP, and JK accomplished genetic and molecular analyses of transgenic lines. RL and SP carried out microscopic studies of transgenic lines. SP performed callose studies. RL, SP, MMH, and JK conceived of this study. RL, SP, and UW drafted the manuscript. All authors read and approved the final manuscript.
